# Influence of Pd, Pt and Au nanoparticles in the photocatalytic performance of N-TiO_2_ support under visible light

**DOI:** 10.1098/rsta.2023.0271

**Published:** 2024-09-23

**Authors:** J. C. Medina, Eleanor Warren, David Morgan, Isla E. Gow, Jennifer Edwards

**Affiliations:** ^1^ Cardiff Catalysis Institute, Cardiff University School of Chemistry, Translational Research Hub, Cardiff University, Maindy Road, Cardiff CF24 4HQ, UK

**Keywords:** water pollution, visible light absorption, metallic nanoparticles

## Abstract

In this article, we report the modification and photocatalytic evaluation of commercial TiO_2_-P25 under visible light for methyl orange (MO) dye degradation under visible light. The activity of materials doped with N, Pd, Pt and Au on to the TiO_2_-P25 was evaluated, with optimal photocatalytic performance achieved using Au nanoparticles doped on an N-functionalized titania surface. X-ray diffraction (XRD), physical nitrogen adsorption/desorption isotherm curves, transmission electron microscopy (TEM), diffuse reflectance spectroscopy, scanning electron microscopy (SEM) and energy dispersive X-ray spectroscopy (EDX) were used to study the structural and textural properties of the samples. The chemical species present in the bulk and surface of the catalysts were identified using X-ray photoelectron spectroscopy (XPS) and microwave plasma-atomic emission spectroscopy. The results show that Au/N-TiO_2_ photocatalyst presents a remarkable enhanced activity for MO dye degradation, under visible light illumination, reaching 100% after 4 h. The enhanced photocatalytic activity using this composite is attributable to the well-dispersed and small size of Au nanoparticles, large surface area, reduction of band-gap energy and the interaction between nitrogen and Au which promoted a synergistic effect.

This article is part of the discussion meeting issue ‘Green carbon for the chemical industry of the future’.

## 1. Introduction

A viable solution providing a renewable and sustainable energy source with the potential to meet continually growing energy demands, as well as the remediation of environmental pollutants can be achieved using photocatalysis. Synthetic dyes are widely employed in the textile industry, posing a significant environmental challenge when disposed of into effluents. Because synthetic dyes are complex, traditional biological treatments cannot effectively achieve both discolouration and wastewater degradation. In recent decades, there has been a surge of interest in advanced oxidation processes as a more efficient approach for eliminating aqueous and gaseous pollutants. Numerous studies have highlighted the photocatalytic activity of oxide semiconductors for oxidizing organic compounds in residual water. Since the seminal work of Fujishima & Honda [1], discussing hydrogen-photo-electrochemical production, titanium dioxide has been extensively utilized as photocatalyst, thanks to its chemical stability, durability, minimal toxicity and favourable production capacity. Furthermore, the high density of states in its bands allows TiO_2_ to efficiently convert photons to current, thereby enhancing its photocatalytic activity beyond that of other semiconductors [2]. However, for its large band gap of 3.2 eV, a TiO_2_ photocatalyst is only effective when irradiated by UV light (comprising only 5% of the solar spectrum), but the most significant impediment to its practical deployment is the quick recombination of its photogenerated electron–hole pairs [3]. This has resulted in many approaches to improve the catalytic activity, including the addition of metal-oxide heterojunctions [4], non-metal element doping [5] and noble-metal doping [6]. Furthermore, it has been demonstrated that a number of variables, including the concentration of the photocatalyst, the pH of the dye solution, the presence of oxidants and the light intensity, affect how quickly azo dyes photodegrade [7]. Therefore, modification of pH, addition of inorganic oxidants [8] (O_3_ or H_2_O_2_) or use of powerful UV light illumination sources are procedures commonly employed to obtain high degradation efficiencies.

The use of noble metals (Au, Ag, Rh and Pd) has shown to be an effective way to change physical parameters such as absorption edge, crystallite size, surface area and charge transfer process. Doping the surface of TiO_2_ with noble metals such as Ag [9], Au [10–12], Pt [13] and Pd [14,15] has been largely investigated by several researchers and shown to increase photocatalytic activity under visible light by acting as an electron trap, thereby augmenting the rates of photo-induced electron transfer at the interface. Additionally, they play a role in inhibiting the recombination of electron–hole pairs. The Fermi levels associated with TiO_2_ are higher than those for noble metals, and significant reductions in electron–hole recombination rates are seen by incorporating TiO_2_ with noble-metal dopants. Kim *et al*. [13] showed that photogenerated electrons become trapped upon modifying TiO_2_ with Pt, thus promoting interfacial charge transfer rates. Seery *et al*. [16] reported that Ag-modified TiO_2_ can extend the photo response of TiO_2_ towards visible light. During dye degradation studies, the addition of Ag was found to enhance the degradation rates of rhodamine 6G dye. In the same study, Ag^+^ ions were found to enhance the visible light absorption capacity of the TiO_2_ material by limiting the recombination rates of photogenerated electrons/holes. Camposeco *et al*. [17] reported a remarkable enhancement of photocatalytic activity in the water-splitting reaction to produce H_2_, where the presence of metallic nanoparticles improved the photocatalytic activity of TiO_2_ nanotubes. Recently, Motamedisade *et al.* [18] presented a novel catalyst for dye degradation fabricated with Au nanoclusters on nitrogen-functionalized TiO_2_. Clark *et al*. [19] have improved the selectivity of methyl orange (MO) and 4-chlorophenol degradation relative to TiO_2_ by using a distorted perovskite structure, CaCu_3_Ti_4_O_12_. The use of square planar Cu^2+^ and octohedral Ti^4+^ sites generates two individual band gaps. Recombination rate is decreased because an electron–hole trap is formed between localized Cu^+^ sites and the conduction band in Ti^3+^.

On the other hand, nitrogen-doped titania has gained significant attention in the field of photocatalysis. Numerous studies have identified it as a promising material for various environmental applications [20–22]. Characteristic features of nitrogen atoms, such as small ionization energy, similar atomic size and high stability compared to oxygen, encourage the incorporation of nitrogen dopants into the structure of TiO_2_ [22,23]. Upon incorporation of nitrogen impurities into the TiO_2_ material, the energy associated with oxygen vacancies decreases from 4.2 to 0.6 eV, thus nitrogen dopants favour oxygen vacancy formation [24]. This doping enhances the material’s photocatalytic efficiency by narrowing the band gap, allowing better utilization of visible light. Furthermore, nitrogen attached to the surface can serve as active sites, forming strong complexes between metallic nanoparticles and titania surfaces. This interaction increases the loading level of the nanoparticles and reduces their agglomeration [18], thereby enhancing the overall photocatalytic efficiency and stability of the material.

It is clear that the use of non-metals and metals on the surface of titania helps to enhance its photocatalytic performance in photocatalytic reactions. In this article, we show the synthesis of modified TiO_2_ photocatalysts for MO dye degradation. The aim was to evaluate the effect of three different metals on nitrogen-functionalized TiO_2_ support (Pd/N-TiO_2_, Pt/N-TiO_2_ and Au/N-TiO_2_) and its influence on the MO dye degradation under visible light illumination.

## 2. Experimental details

### (a) Photocatalyst synthesis

#### (i) N-doped TiO_2_ support

Hexamethylenetetramine (HMT), C_6_H_12_N_4_(Sigma-Aldrich) was used as the nitrogen source. Manual grinding of TiO_2_ powder P25 (Degussa) and HMT was performed for 15 min using a pestle and mortar. The amount of HMT was 20 wt% and the sample was identified as N-TiO_2_. The resultant N-doped TiO_2_ was calcined at 400°C for 1 h in flowing air with a ramp rate of 5°C min^−1^.

#### (ii) Addition of metallic nanoparticles to N-TiO_2_ support

Gold, platinum and palladium nanoparticles were added to the N-doped TiO_2_ support by deposition–precipitation with the urea (DPU) method in the absence of light. The target metal concentration was 1 wt%. The metal precursors were hydrogen tetrachloroaurate (III) hydrate (HAuCl_4_·3H_2_O), potassium tetrachloroplatinate (K_2_PtCl_4_), palladium nitrate hydrate Pd(NO_3_)_2_2H_2_O and urea (0.42 M), all supplied by Sigma-Aldrich. In all cases, the metal precursor and urea were dissolved in 20 ml of distilled water. Then, 500 mg of N-TiO_2_ (obtained by manual grinding) was added to the solution; subsequently, the suspension was vigorously stirred at 80°C for 16 h.

After the DPU method was completed, all materials were filtered, washed with distilled water and dried at 80°C for 12 h. Following drying, the samples were vacuum-sealed and kept at room temperature in a desiccator, shielded from light, to prevent any alterations.

All samples underwent thermal activation treatments under H_2_ at 400°C for 2 h with a ramp rate of 5°C min^−1^.

### (b) Characterization techniques

The crystalline structure of the materials was studied using X-ray diffraction (XRD) and a PANalytical X’pert Pro powder diffractometer operating at 40 kV, 40 mA using Cu Kα radiation (*λ* = 1.54 × 10^–10^ m) with a Ge (111) single crystal monochromator. The phases present were assigned using the International Centre for Diffraction Data database as a reference. Transmission electron microscopy (TEM) analysis was conducted using a JEOL JEM-2100 operating at 200 kV. Powders were deposited on to 300 mesh copper grids coated with holey carbon film for imaging. Scanning electron microscopy (SEM) was performed on a Tescan Maia3 field emission gun microscope. An Oxford Instruments XMAX^N^ 80 energy dispersive X-ray (EDX) detector was used for elemental analysis, controlled by Aztec software. The samples were dispersed as powders on to adhesive carbon Leit discs fixed to 12.5 mm aluminium stubs. For imaging purposes, the samples were coated with a 10 nm coating of 80:20 Au:Pd atoms by a Quorum plasma coater to prevent charging, and images were acquired using the secondary electron detector. The uncoated samples were analysed for EDX using the backscattered electron detector. The optical properties of the photocatalysts were measured using a UV–vis spectrometer, CARY 4000, equipped with a Harrick Praying Mantis attachment.

The textural properties were obtained using a Quantachrome Nova 2200E. A Brunauer–Emmett–Teller (BET) equation based on N_2_ physisorption data was used to determine the specific surface area. The Barrett–Joyner–Halenda method was applied to calculate the pore size distribution from the desorption branch of the isotherm. The materials were dried for 4 h at 100°C and outgassed prior to nitrogen adsorption. The chemical composition of the materials was analysed on a Thermo Fisher Scientific K-Alpha^+^ photoelectron spectrometer utilizing micro-focussed monochromatic Al Kα radiation operating at 72 W power (6 mA × 12 kV), utilizing the 400 micron spot mode, which affords an elliptical analysis area of *ca* 400 x 600 microns. Data were calibrated to the Ti 2p peak for the TiO_2_ support (taken to be 458.8 eV) using an approach that avoids the uncertainties in calibration to the C1s signal, which can be affected by changes in the surface chemistry. Data were analysed using CasaXPS (v2.3.26 PR1.0N) [25]. Surface homogeneous equivalent concentrations were calculated using transmission-corrected spectra, with a combination of Scofield sensitivity factors and an electron escape depth according to the TPP-2M formulation after Shirley background subtraction. The chemical content of Au, Pd and Pt in the dried samples was achieved by microwave plasma-atomic emission spectroscopy (MP-AES). The metal catalysts were filtered and diluted to a metal concentration of approximately 10 ppm after being digested for 24 h (50 mg of catalyst and 5 ml of aqua regia). Metal concentrations were measured using an Agilent 4200 MP-AES 7900. Grams of metal per gram of material were used to express the metal weight loadings.

### (c) Photocatalytic activity evaluation using visible light

The photocatalytic activity evaluation of the samples was performed in an open-air, glass photoreactor system containing 0.025 g of the catalyst and 50 ml of an aqueous solution of MO dye (10 ppm). The exposed-to-air suspension was kept at room temperature with no pH change (pH = 7), and with magnetic stirring at 350 r.p.m. A cold water bath was used to reduce thermal effects maintaining a temperature range between 25 and 30°C. The resultant solution was irradiated with visible light (*λ* > 380 nm; 68 mW cm^−2^) using a SOLIS-3C high-power LED lamp (Thorlabs) over 4 h. To guarantee the adsorption–desorption equilibrium, the suspension was held in the dark for 30 min prior to being exposed to visible light. The method used to measure the MO dye concentration in each case was to acquire a filtered aliquot of one millilitre at regular intervals, using a UV–vis spectrophotometer to measure the aliquot absorbance (with the MO dye absorption band at 464 nm).

Percentage degradation = (1 *C*/*C*_o_) × 100, where the MO solution absorbance maxima before and after the radiation period are denoted by *C*_o_ and *C*, respectively. The Langmuir–Hinshelwood kinetic model, which is typically used to describe the photodegradation kinetics on semiconductors, was used to determine the reaction rate constant. Pseudo-first-order kinetics with an apparent first-order rate constant (*k*_app_) can be used to simplify the reduction rate (*r*) in cases where the adsorption or reactant concentration is low. The initial concentration (*C*_*o*_) is the reactant concentration after reaching time (*t*), which can be expressed as


$$\text{ln}(C/C_{o})=-k_{r}k_{ads}C=-k_{app}t,$$

where *k*_*r*_ is the rate constant and *k*_ads_ is the adsorption equilibrium constant. The apparent rate constant (*k*_app_) is the slope of a straight line obtained by plotting ln(*C*_o_/*C*) against reaction time (*t*).

## 3. Results

### (a) Structure of the photocatalysts

**Figure 1.**
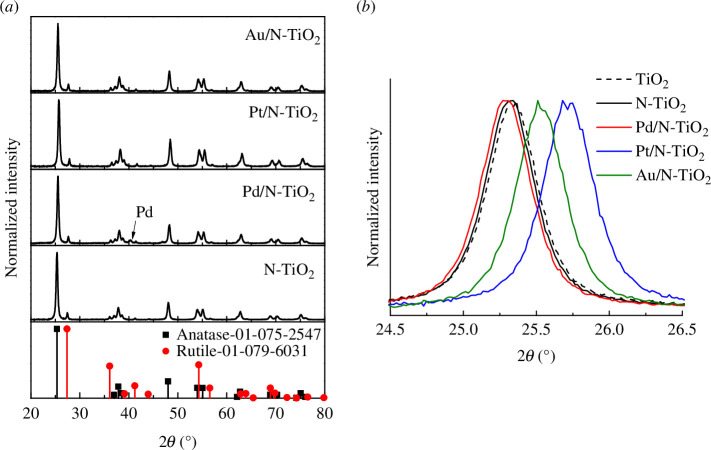
XRD pattern of (*a*) N-TiO_2_, Pd/N-TiO_2_, Pt/N-TiO_2_ and Au/N-TiO_2_ catalysts. (*b*) Magnified XRD peak at 25.3^◦^ (101) for all catalysts.

Figure 1 illustrates the XRD patterns of N-doped support decorated with Pd, Pt and Au. They were all showing signs the presence the anatase polymorph with reflections at 25.3° (101), 36.9° (103), 37.8° (004), 38.6° (112), 48.1° (200), 53.9° (105), 55.1° (204), 62.14° (213), 62.7° (204), 68.8° (116), 70.30° (220), 75.0° (215) and 76.0° (301), agreed with the JCPDS 01-075-2547 card; also the reflections related to the rutile phase at 27.4° (110), 36.1° (101), 41.3° (111) and 44.1° (210) agreed with the JCPDS 01-079-6031 card. No evidence of nitrogen was observed in the N-TiO_2_ pattern. However, in the Pd/N-TiO_2_ pattern, a reflection related to Pd (111) was identified according to matching JCPDS 01-087-0645. The absence of reflections correlating to Pt and Au phases in the XRD pattern of Pt/N-TiO_2_ and Au/N-TiO_2_ indicates that these metal particles are too small to be detected on the surface of N-TiO_2_ surface. The crystallite size of the TiO_2_ in each catalyst was determined using the Scherrer equation at 2*θ* = 25.3° (101) for the anatase phase and 2*θ* = 27.4° (110) for the rutile phase. The findings indicate that the addition of metallic nanoparticles on the surface of N-TiO_2_ did not drastically affect the crystallite size for either phase, when compared to pristine P25 TiO_2_, with the exception of Au/N-TiO_2_ catalyst for rutile phase (see table 1). Figure 1*b* shows that the XRD reflections at 2*θ* = 25.3° for Pt and Au/N-TiO_2_ catalyst are shifted to larger angles in comparison with pristine TiO_2_. Table 1 indicates that for the materials, the measured loadings by MP-AES of Pd, Pt and Au metals were quite similar to the nominal one (1 wt%), indicating that DPU has good metal deposition efficiency.

**Table 1.** Textural properties of the compounds and their metal load.

catalyst	*S*_BET_ (m^2^g^−1^)	average pore size (Å)	crystallite size of anatase (nm)	crystallite size of rutile (nm)	anatase (101) peak position (2θ °)	*d*-spacing (nm)	actual *M* (*M* = Pd, Pt or Au) content wt%
TiO_2_	54	45.1	21	31	25.3	35.1	—
N-TiO_2_	56	54.9	21	30	25.3	35.1	—
Pd/N-TiO_2_	48	17.9	21	31	25.3	35.2	1.2
Pt/N-TiO_2_	63	141	20	32	25.7	34.6	1.1
Au/N-TiO_2_	95	162	21	35	25.5	34.8	1.1

Metal loading determined by MP-AES.

### (b) Textural properties

**Figure 2.**
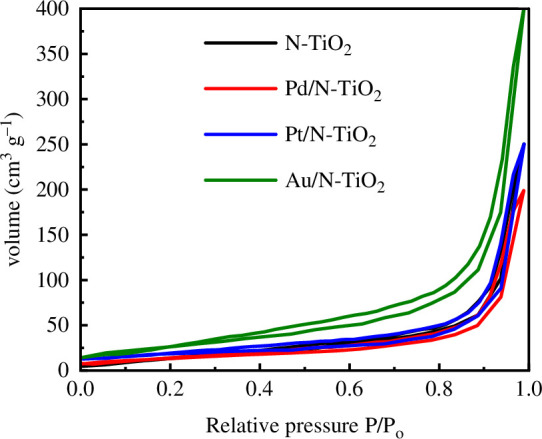
N_2_ adsorption/desorption isotherms of N-TiO_2_, Pd/N-TiO_2_, Pt/N-TiO_2_ and Au/N-TiO_2_ catalysts.

The values of the specific surface areas for N-TiO_2_, Pd/N-TiO_2_, Pt/N-TiO_2_ and Au/N-TiO_2_ catalysts were 56, 48, 63 and 95 m^2^ g^−1^, respectively. The specific surface area of N-TiO_2_ did not drastically change, while for the Pd/N-TiO_2_ catalyst it decreased with respect to pristine TiO_2_-P25 (54 m^2^ g^−1^), probably as a result of Pd deposition causing partial occlusion of the support’s pores. On the other hand, the deposition of Pt and Au increased the specific surface area of these compounds compared to the N-TiO_2_ support, which was more evident in the Au/N-TiO_2_ catalyst. The findings of the adsorption/desorption analysis for N-TiO_2_, Pd/N-TiO_2_, Pt/N-TiO_2_ and Au/N-TiO_2_ are shown in figure 2. All of the photocatalysts presented N_2_ adsorption/desorption isotherm curves type IV with an H3-type hysteresis loop, indicating a distinctive mesoporous structure. The average pore sizes for the N-TiO_2_, Pd/N-TiO_2_, Pt/N-TiO_2_ and Au/N-TiO_2_ catalysts were 54.9, 17.9, 141 and 162 Å, respectively. A general trend was observed, with samples having a larger specific surface area also showing a larger pore size, probably owing to the influence of pH on the etching N-TiO_2_ surface during the deposition precipitation method using urea [26,27].

### (c) TEM and XPS

**Figure 3.**
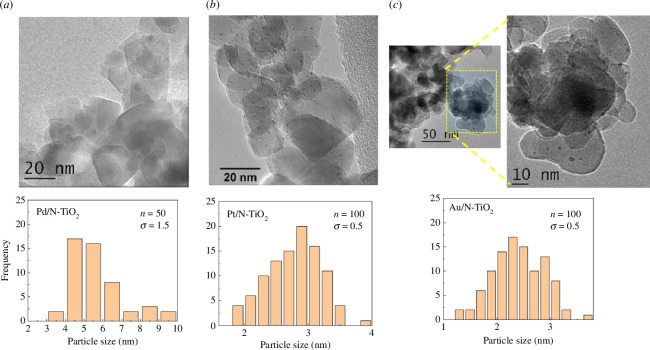
TEM images and particle size distribution for (*a*) Pd/N-TiO_2_, (*b*) pt/N-TiO_2_ and (*c*) Au/N-TiO_2_.

Figure 3*a–c* shows the TEM images and the associated dispersion of metal particle sizes for Pd/N-TiO_2_, Pt/N-TiO_2_ and Au/N-TiO_2_ catalysts. Semi-spherical metallic nanoparticles of Pt and Au were well-dispersed on the N-TiO_2_ surface with an average diameter of 3 and 2 nm, respectively. In contrast, the metallic nanoparticles of Pd were not as well dispersed on the support surface, with an average diameter of 6 nm and with a higher standard deviation (1.5) with respective to Pt and Au nanoparticles (0.5). These observations of particle size are in line with results from XPS, where the full-width-half-maximum of the nanoparticles are *ca.* 0.3–0.4 eV broader than their bulk metal equivalents (recorded under identical conditions) and have some differences in peak asymmetry for the Pt and Pd nanoparticles [28–30].

**Figure 4.**
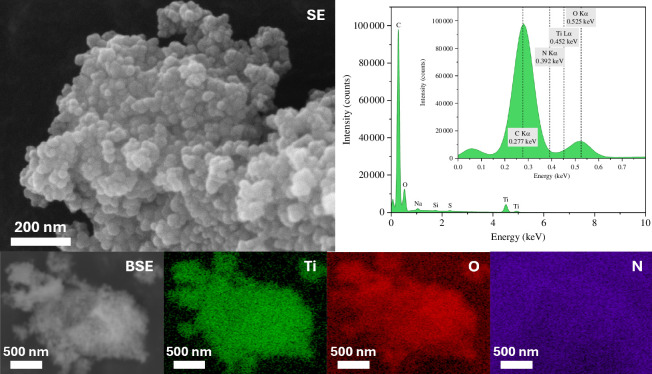
SEM imaging and EDX mapping of N-TiO_2_. Secondary electron image taken at 250 kx. Backscattered electron image taken at 41 kx. Elemental maps show titanium, oxygen and nitrogen in area of BSE image, with EDX spectrum showing intensities of the elements detected.

**Figure 5.**
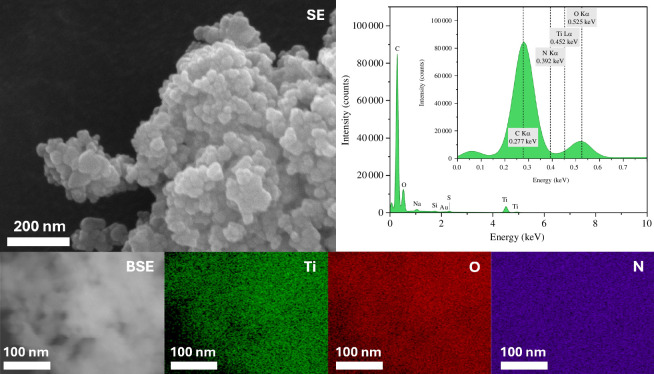
SEM imaging and EDX mapping of Au/N-TiO_2_. Secondary electron image taken at 250 kx. Backscattered electron image taken at 320 kx. Elemental maps show titanium, oxygen and nitrogen in area of BSE image, with EDX spectrum showing intensities of the elements detected.

The surface elemental composition of the photocatalysts was studied using XPS to determine the extent of surface dopant inclusion. Table 2 lists the values for all the chemical elements present on the surface of the catalysts. In each case, nitrogen was observed. SEM-EDX was used to further investigate the existence of nitrogen in the catalysts. In contrast to the findings from XPS, nitrogen was not clearly observed by EDX in either N-TiO_2_ (figure 4) or Au/N-TiO_2_ (figure 5); however, this is believed to be due to the close overlap in the energies of the Kα peaks for carbon (present from the adhesive carbon discs) and nitrogen, at 0.277 keV and 0.392 keV, respectively (see inset EDX spectrum). The presence of S is also explained by the adhesive used in the carbon discs, and Si is an artefact of the silicon drift detector used in EDX.

**Table 2.** Surface elemental composition of all catalysts as determined by XPS.

sample	C 1s (% atom)	N 1s (% atom)	O 1s (% atom)	Ti 2p (% atom)	Pd 3d (% atom)	Pt 4f (% atom)	Au 4f (% atom)
N-TiO_2_	27.9	0.29	50.67	21.13	—	—	—
Pd/N-TiO_2_	25.39	0.26	52.07	21.99	0.15	—	—
Pt/N-TiO_2_	26.04	0.19	51.78	21.67	—	0.18	—
Au/N-TiO_2_	25.74	0.26	52.27	21.41	—	—	0.18

### (d) Diffuse reflectance spectroscopy (DRS)

**Figure 6.**
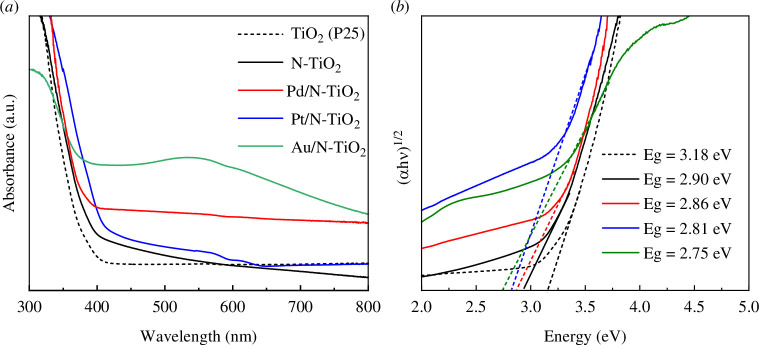
*(a*) Diffuse reflectance spectra and (*b*) diffuse absorbance spectra of the (αhν)^1/2^ plots for N-TiO_2_, Pd/N-TiO_2_, Pt/N-TiO_2_, Au/N-TiO_2_ and TiO_2_-P25.

Figure 6*a* shows the results of DRS of N-TiO_2_, Pd/N-TiO_2_, Pt/N-TiO_2_ and Au/N-TiO_2_ catalysts. The DRS of commercial TiO_2_-P25 was also included as reference material. The band-gap energies of 3.2 and 3.0 eV, respectively, are associated with the well-known absorption of TiO_2_ anatase and rutile phases, which are, respectively, approximately 387 and 413 nm in wavelength [31,32]. In comparison to TiO_2_-P25, all of the surface-modified composites exhibited a shift in absorption to longer wavelengths, which was caused by the extra energy levels produced by N, Pd, Pt and Au on the surface of pristine TiO_2_-P25. Additionally, the surface plasmon resonance (SPR) in the Au/N-TiO_2_ photocatalyst reveals a unique behaviour concerning the absorption of gold nanoparticles in the visible spectrum (500−600 nm), verifying that the Au nanoparticles achieved the zero-valence state after being thermally treated in an H_2_ environment [33]. The SPR phenomenon of metallic nanoparticles is dependent on several parameters, including the size, shape, loading and dielectric characteristics of the surrounding medium [34,35]. Optical band-gap (*E*_*g*_) energies obtained from diffuse reflectance spectra are shown in figure 6*b*. The addition of nitrogen on the TiO_2_-P25 surface helps to decrease its band gap from 3.18 to 2.9 eV. For Pd-, Pt- and Au-decorated photocatalysts, the reduction in band-gap energies was notably larger, measured at 2.86, 2.81 and 2.75 eV, respectively.

### (e) Photocatalytic activity evaluation

**Figure 7.**
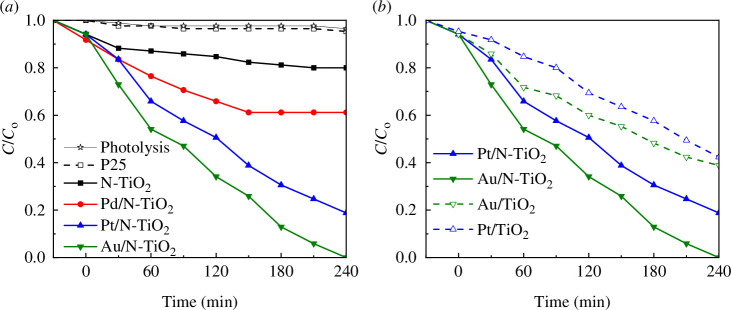
Evaluation of photocatalytic activity for (*a*) N-TiO_2_, Pd/N-TiO_2_, Pt/N-TiO_2_, Au/N-TiO_2_, TiO_2_-P25 and (*b*) photocatalytic evaluation of compounds with and without nitrogen.

Figure 7*a* shows the relative concentration profile (*C*/*C*_o_) of the MO dye during both the photocatalytic and photolysis processes for TiO_2_-P25, N-TiO_2_, Pd/N-TiO_2_, Pt/N-TiO_2,_ and Au/N-TiO_2_ materials. The *C*/*C*_o_ graphs show the initial dye concentration (*C*/*C*_o_ = 1), the data were obtained after 30 min in darkness with stirring (*t* = 0), and the subsequent values are post-illumination because, as was previously noted, the adsorption–desorption equilibrium was reached after 30 min.

Under the visible light illumination conditions applied in this study, it is evident that the dye concentration remained unchanged during dark conditions, indicating negligible adsorption of the dye on to the powder surfaces. Furthermore, the photolysis exhibited only marginal decreases in the *C*/*C*_o_ ratio, confirming the stability of the MO dye throughout the irradiation. This observation, coupled with the absence of dye adsorption on the powder surfaces and minimal photolysis, allows us to classify the decrease in time of the *C*/*C*_o_ ratio as a discolouration process caused by photocatalytic activity in semiconductor materials, which is not the same as mineralization of the dye. Mineralization refers to the complete conversion of dye molecules to inorganic substances such as CO_2_ and H_2_O.

In the photocatalytic reaction using the TiO_2_-P25, it was observed that the *C*/*C*_o_ of the dye is very similar to the photolysis, which was expected owing to the value of its band gap (3.18 eV). In addition, the N-TiO_2_ powder achieved a notable discolouration of the MO dye after 4 h. Incorporating Pd, Pt and Au nanoparticles on to the surface of N-TiO_2_ notably enhanced its photocatalytic performance under visible light, culminating in complete discolouration of the MO dye by Au/N-TiO_2_ within 4 h. Furthermore, as shown figure 7*b*, the photocatalytic activity of Pt/N-TiO_2_, Au/N-TiO_2_, Pt/TiO_2_ and Au/TiO_2_ indicates that the combination of nitrogen and metallic nanoparticles on the TiO_2_-P25 surface exhibited greater efficiency compared to the presence of metallic nanoparticles alone. This indicates a synergistic effect between the N and the Au/Pt. The apparent kinetic rate constants (*K*_app_) calculated using the pseudo-first-order model for N-TiO_2_, Pd/N-TiO_2_, Pt/N-TiO_2_ and Au/N-TiO_2_ were 0.0007, 0.0028, 0.0054 0.0083 min^−1^, respectively.

**Figure 8.**
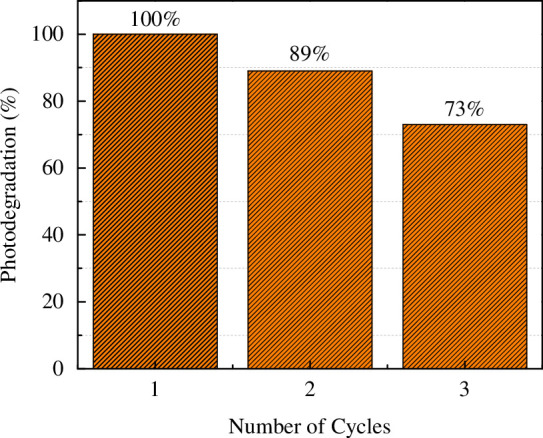
Percentage degradation values of MO dye obtained from the stability test using Au/TiO_2_ catalyst.

The stability of the best catalyst (Au/N-TiO_2_) was tested by performing three consecutive cycles for MO dye degradation. The percentage value of MO dye degradation obtained in each cycle is shown in figure 8. The sample was gathered and centrifuged following each cycle to assess its potential for reuse. The photodegradation efficacies of MO dye were 99.9, 89 and 73% for first, second and third cycles, respectively. The photodegradation effectiveness of the Au/N-TiO_2_ catalyst was diminished as the cycle number increased; powder loss during suspension and centrifugation treatment may be the primary cause of this.

## 4. Discussion

In this work, nitrogen and metallic nanoparticles (Pd, Pt and Au) were used to modify commercial TiO_2_-P25 and to improve its photocatalytic performance under visible light to degrade the MO dye. Interestingly, these combinations (metal and non-metal) have been used to enhance visible light absorption of TiO_2_ previously [6]. The findings of this investigation reveal that the best photocatalyst for MO dye degradation under visible light illumination was the Au/N-TiO_2_ composite, followed in effectiveness by the Pt/N-TiO_2_ composite, with the least effective catalyst being the Pd/N-TiO_2_ composite.

The improved photocatalytic activity, using visible light illumination, of the Pt/N-TiO_2_ and Au/N-TiO_2_ photocatalysts is associated with their fundamental physical characteristics. It is commonly recognized that photocatalysts with large surface areas and/or small crystal sizes exhibit enhanced photocatalytic performance when it comes to the breakdown of organic compounds. The results demonstrate that the inclusion of all dopants on the TiO_2_-P25 increased the surface area in all cases except for palladium. The largest increase in surface area was from 54 to 95 m^2^ g^−1^ for Au/N-TiO_2_, while the crystalline sizes did not change with the addition of N, Pd, Pt or Au with respect to bare TiO_2_. Furthermore, the increased size and well-defined mesoporous structure of the Pt and Au catalysts enabled MO molecules (6–8 nm) to transfer and diffuse into the interior pores (14.1 nm for Pt/N-TiO_2_ and 16.2 nm for Au/N-TiO_2_), thus increasing the MO-photocatalyst contact interface and providing more reactive sites, resulting in an increase of MO dye degradation. The majority of previous research has concentrated on metallic nanoparticles with sizes ranging from 5 to 100 nm and found that as size decreased, catalytic activity increased [36–42]. On a few occasions, nanoparticle catalytic activity may decrease with decreasing size or remain unchanged [43,44]. It has been shown that small gold nanoclusters (<1.5 nm) exhibit a quantized electronic structure similar to a molecule owing to the quantum confinement phenomenon [45,46]. In this study, the nanoparticle sizes obtained were 5, 3 and 2 nm for Pd, Pt and Au, respectively.

All the diffraction patterns exhibited both the anatase and rutile phases related to commercial TiO_2_-P25 except the Pd/N-TiO_2_ material, where one small reflection was observed indicating the presence of metallic Pd. This observation suggests a larger particle size for Pd nanoparticles compared to the Pt and Au. Figure 1*b* shows that in comparison to TiO_2_-P25, the XRD reflections of Pt/N-TiO_2_ and Au/N-TiO_2_ are displaced to higher angles, indicating a lattice contraction. The synergy observed between Pt and Au ions with TiO_2_ is undoubtedly crucial, given that doped titania reveals diverse benefits to its photocatalytic activities by modifying its surface features and reducing the band gap. This agrees with the estimated optical band-gap (*E*_*g*_) energies obtained from DRS for all materials that suffered a decrease with respect to *E*_*g*_ values for bare TiO_2_. Specifically, Ti 3d and O 2p orbitals constitute the majority of the conduction and valence bands in the TiO_2_ matrix [47,48]. Introducing additional metal ions into the structure of TiO_2_ leads to interactions between the outer shell orbitals of these ions and the energy states in the bands, resulting in the creation of impurity levels and alterations in the band structures. Indeed, the influence of dopants on the electronic structures of semiconductors is closely linked to several factors, including the dopant atomic numbers, ionic radii and oxidation states [2,17,48]. These characteristics determine the extent of modifications in the band structure, impurity levels and, ultimately, the photocatalytic performance of the doped material. In summary, the presence of Pt and Au nanoparticles on the N-TiO_2_ surface leads to a significant distortion of the octahedral geometry, causing the conduction-band minimum to shift downward to lower energy levels, modifying its electronic properties and catalytic behaviour.

The surface chemical composition was obtained using XPS (table 2). It is important to emphasize that in each case nitrogen was detected on the surface of the TiO_2_-P25, and so we propose the near-surface is N functionalized, which, with a nitrogen binding energy of 399.7 eV, is characteristic of NH_x_ species; however, the insertion of nitrogen at interstitial sites cannot be excluded [49]. Studies have demonstrated that co-catalysts (metallic nanoparticles) and N-functionalized TiO_2_ surfaces can form strong complexes with N bound on the surface, increasing loading levels and reducing co-catalyst agglomeration [18,50–52]. This can be seen in figure 5*b*, where the photocatalytic activity of the composites with N and Pt or Au nanoparticles presents much better photocatalytic performance than the composites with only Pt or Au nanoparticles on the surface, suggesting a synergistic effect between N and metallic nanoparticles.

There are some reports related to the use of non-metal and metal ions to improve the photocatalytic efficiency of TiO_2_ for dye degradation [4,53,54]. Nonetheless, the Au/N-TiO_2_ photocatalyst prepared in this study showed comparable or even better photocatalytic performance for MO dye degradation than those reported in previous works (table 3) without altering the pH, adding H_2_O_2_ or using UV illumination. The apparent kinetic rate constant (*k*_app_) for the Au/N-TiO_2_ photocatalyst was 0.0083 min^−1^, which is more than ten times larger than the *K*_app_ of N-TiO_2_ composite (0.0007 min^−1^), two times larger than that estimated by Kader *et al*. [59] (0.004 min^−1^) and more than five times larger than the one reported by Ellouzi *et al*. [53] (0.0015 min^−1^) under similar experimental conditions. However, it is important to mention that the lack of standardization makes it difficult to reliably compare and reproduce findings across photocatalytic studies. Kisch & Bahnemann [64] suggest that for solid/liquid photocatalytic systems, an apparent optimal quantum yield should be obtained, which improves the comparability of photocatalytic activity results across different laboratories.

**Table 3.** A comparison of the photocatalytic activity between the current study and other published works.

material	dye	catalyst amount (g)	dye conc. (ppm)	pH	type of light	illumination time (min)	effectiveness (%)	ref.
TiO_2_ NPsMO	MO	0.15	15	3	100 W UV lamp	300	99.8	[55]
TiO_2_ NPs	MO	0.10	5	—	182 W UV lamp	240	41.1	[56]
TiO_2_ spheres	MO	0.02	10	7	32 W Hg lamp	140	89.7	[57]
TiO_2_/ASS	MO	0.2	25	7	UV lamp	360	90	[58]
Ag/MoO_3_/TiO_2_	MO	0.12	10	7	UV irradiation	330	75.8	[59]
C, N, S–Fe–TiO_2_	MO	0.5	10	—	6 W visible lamp	180	25	[53]
M/TiO_2_ (*M* = Mn, Ni, Co)	MO	—	8	—	300 W lamp that simulates solar radiation	600	60	[60]
NiSO4/TiO_2_	MO	1 g l^−1^	10	5.8	UV irradiation	120	31	[54]
TiO_2_–Ag–WO_3_	methylene blue	0.5	10	7	121 W visible light source	60	72	[61]
TiO_2_–Au–WO_3_	methylene blue	0.02	30	—	300 W xenon lamps	240	94.5	[62]
TiO_2_-F–WO_3_	MO	0.12	10	3	100 W UV irradiation	330	99.68	[63]
Au/N–TiO_2_	MO	0.025	10	7	visible light	240	99.9	this work

## 5. Conclusion

This work demonstrates that doping TiO_2_ with nitrogen and metallic nanoparticles (Pd/N-TiO_2_, Pt/N-TiO_2_ and Au/N-TiO_2_) enhances the photocatalytic decomposition of MO mediated by visible light. The optimum photocatalytic activity in MO dye degradation was achieved using Au/N-TiO_2_ photocatalyst with 99.9% degradation observed after 4 h with an apparent first-order constant rate of *k*_app_ = 0.0083 min^−1^. The photocatalytic performance during visible light illumination of Au/N-TiO_2_ with respect to the other composites is explained by the well-dispersed and smaller nanoparticle size (2 nm), largest surface area (95 m^2^g^−1^, 1.7 times larger than bare TiO_2_), lowest band-gap energy (2.75 eV) and synergistic effect at the interface sites between N and Au nanoparticles, with all of these confirmed by XRD, TEM, BET, DRS and XPS characterization techniques.

## Data Availability

Information on the data underpinning the results presented here, including how to access them, can be found in the Cardiff University data catalogue at [65].
